# Peri-implant bone regeneration in pigs

**DOI:** 10.1186/s40729-024-00572-9

**Published:** 2024-11-15

**Authors:** Siddharth Shanbhag, Javier Sanz-Esporrin, Carina Kampleitner, Stein-Atle Lie, Reinhard Gruber, Kamal Mustafa, Mariano Sanz

**Affiliations:** 1 Department of Immunology and Transfusion Medicine, Haukeland University Hospital, Bergen, Norway; 2 Center for Translational Oral Research (TOR), Department of Clinical Dentistry, Faculty of Medicine, University of Bergen, Bergen, Norway; 3 Department of Periodontology, Faculty of Dentistry, University of Oslo, Oslo, Norway; 4 ETEP Research Group, Faculty of Odontology, University Complutense of Madrid, Madrid, Spain; 5 Karl Donath Laboratory for Hard Tissue and Biomaterial Research, Division of Oral Surgery, University Clinic of Dentistry, Medical University of Vienna, Vienna, Austria; 6 Ludwig Boltzmann Institute for Experimental and Clinical Traumatology, The Research Center in Cooperation with AUVA, Vienna, Austria; 7 Austrian Cluster for Tissue Regeneration, Vienna, Austria; 8 Department of Oral Biology, University Clinic of Dentistry, Medical University of Vienna, Vienna, Austria; 9 Department of Periodontology, School of Dental Medicine, University of Bern, Bern, Switzerland

**Keywords:** Bone regeneration, Peri-implantitis, Animal models, Systematic reviews

## Abstract

**Purpose:**

To review the current literature to answer the focused question: in the experimental pig model (population), which types of peri-implant bone defects (exposure) have been used evaluate different modes of therapy and what is their capacity for spontaneous healing and regeneration (outcome)?

**Methods:**

Following PRISMA guidelines, electronic databases were searched for studies reporting peri-implant bone defects in the maxillae or mandibles of pigs. Those studies which reported a control group of untreated defects with assessment of spontaneous regeneration [new bone area (BA)] and/or re-osseointegration [new bone-to-implant contact (BIC)] via quantitative radiography or histomorphometry were included in a random effects meta-analysis for the outcomes BA and BIC.

**Results:**

Overall, 21 studies, mostly performed in the mandibles of minipigs, were included. Most studies reported ‘acute’ intrabony (circumferential and/or dehiscence; *n* = 12) or supra-alveolar defects (horizontal; *n* = 4). Five studies attempted to induce ‘chronic’ peri-implantitis lesions using ligatures with conflicting results. Meta-analyses revealed pooled estimates (with 95% confidence intervals) of 48.07% BIC (30.14–66%) and 64.31% BA (42.71–85.91%) in intrabony defects, and 52.09% BIC (41.83–62.35%) and 28.62% BA (12.97–44.28%) in supra-alveolar defects. Heterogeneity in the meta-analysis was high (I^2^ > 90%).

**Conclusion:**

Current evidence for peri-implant bone regeneration in pigs is mainly based on acute intrabony defects, which demonstrate a high capacity for spontaneous regeneration and re-osseointegration. The evidence for chronic peri-implantitis is limited and does not clearly indicate a spontaneous progression of the disease in this animal model.

**Supplementary Information:**

The online version contains supplementary material available at 10.1186/s40729-024-00572-9.

## Background

Bone regeneration around dental implants as a result of insufficient bony volume or as a treatment of the sequelae from peri-implantitis presents a clinical challenge. These bone defects present a net loss of osseointegration, i.e., diminished direct bone-to-implant contact (BIC), potentially compromising short- and long-term treatment outcomes [1, 2]. The treatment objective herein, beyond the arrest of the inflammation by infection control measures, is to reconstruct the bone architecture around the implant and reestablish the lost BIC [3]. Results from clinical studies have recommended different regenerative therapies for the different peri-implant defects, usually following the principles of guided bone regeneration (GBR), using barrier membranes in combination with bone grafts and/or bone substitute materials [4, 5]. Similarly, there is evidence from preclinical studies on the achieved regenerative outcomes, including the re-establishment of ‘osseointegration’, with the concomitant rise in BIC percentages, even in cases of previously contaminated implant surfaces [6, 7]. Although these regenerative surgical procedures have shown a certain degree of efficacy depending on the defect architecture, there is no consensus on the effectiveness of one technique over the other [8–12].

Preclinical testing of new regenerative therapies in clinically relevant animal models is an important aspect of translational research and, in most cases, a requirement of regulatory health agencies before initiating human clinical trials [13, 14]. In particular, large-animal models (dogs, pigs, sheep, and non-human primates) are used to simulate clinical conditions, and hence, predict therapeutic efficacy and foster human clinical research [14]. Although non-human primates (NHPs) represent the closest animal model to humans, based on genetic background and biological similarity, the economic and ethical concerns surrounding their use have made this model almost completely non-viable in several countries [15]. Hence, dog, sheep, goat, and pig models are the preferred alternatives since their bone composition and biology are similar to those of humans.

From these, dog models are arguably the most frequently used in peri-implantitis research [16, 17]. Preclinical dog models of peri-implantitis are broadly categorized as either the experimental model of ligature-induced “chronic” peri-implantitis (LIPI), or “acute” surgically developed bone defects around implants. Since dogs have a natural susceptibility to periodontal (and peri-implant) diseases, the chronic LIPI model represents the ‘gold standard’ to investigate both the pathogenesis of peri-implant diseases and the efficacy of bone regenerative therapies around implants [18, 19]. However, as for NHPs, the use of dogs in experimental in vivo investigations has raised significant criticisms given their role in society as companion animals. Therefore, there is a growing trend towards the ‘phasing out’ of dog models and the promotion of other animals (e.g. pigs) as the preclinical model of choice in bone regenerative studies.

Conversely to dogs, pigs are considered to be food producing animals, and therefore, their use in experimental in vivo investigations may have the advantage of a relatively less critical public perception. In fact, a recent survey showed that there is a perceived difference in moral status between companion animals and farm animals, such as pigs [20]. The advent of miniature pigs or minipigs, being smaller and easier to manage than domestic breeds, has further contributed to their preference as experimental animals. Additional advantages in their use are their easy availability, relatively low cost, ability to produce large litters, and the possibility to obtain a larger volume of tissue biopsies [21–24]. Furthermore, pigs are closely related to humans in terms of bone anatomy, composition, and metabolism [25–27].

While the possibility of inducing chronic peri-implantitis in pig models has been suggested from as early as 1991 [28], compared to dogs, there is relatively less information on the characteristics of experimental peri-implant bone defects in pigs. It is also presently unclear which defect designs and dimensions in pigs most accurately represent a critical-size defect (CSD) around implants, i.e., the smallest-size experimental defect that will not spontaneously and completely regenerate with bone in a defined timeframe without intervention [29, 30]. In context, we have recently demonstrated through a meta-analysis the relatively high capacity for spontaneous regeneration (~ 40–50%) in experimental alveolar bone defects of pigs [31]. Systematic reviews and meta-analyses of animal studies can be useful for detecting heterogeneity and improving the methodological quality of future studies, allowing for reliable comparisons and more accurate clinical translation [32]. Therefore, our present objective was to systematically review the literature to answer the focused PEO (population, exposure, outcome) question: in the pig model (P), what are the characteristics of experimental peri-implant bone defects (E) in terms of their three-dimensional configuration and capacity for spontaneous regeneration, i.e., new bone formation and new BIC or re-osseointegration (O)?

## Methods

### Study design

The review protocol was based on the Preferred Reporting Items for Systematic reviews and Meta-Analyses (PRISMA) [33] and Systematic Review Centre for Laboratory Animal Experimentation (SYRCLE) guidelines [34], and registered on the database PROSPERO: International Prospective Register of Systematic Reviews (CRD42023450700). The results of studies reporting on alveolar bone defects (without implants) are published elsewhere [31].

Inclusion criteria:

1. Experimental in vivo studies in pigs, including minipigs.
2. Creation of experimental peri-implant bone defects, either chronic (LIPI) or acute (surgically created), in the maxilla or mandible.
3. Quantitative assessment of new bone formation and/or re-osseointegration (new BIC) using clinical measurements, three-dimensional (3D) radiography/tomography [computerized tomography (CT), cone-beam CT (CBCT), micro-CT] and/or 2D histomorphometry.

Exclusion criteria:

1. In vivo studies in other animal species.
2. In vivo studies reporting defects in other anatomical sites (calvarial or non-maxillofacial) and ectopic (subcutaneous or intramuscular implantation) models.
3. Reporting of only qualitative or semiquantitative radiographic and/or histological analyses.
4. In vitro and in silico studies.
5. Clinical studies.

Outcome: The primary outcome of interest was characterizing the different types of peri-implant defects reported in minipigs. The secondary outcome of interest was the amount of unassisted or spontaneous regeneration (new bone formation) and reosseointegration (BIC) in untreated control defects assessed by 3D tomography or 2D histomorphometry.

### Search strategy, screening, and study selection

A search strategy was developed with assistance from the University of Bergen library in accordance with the SYRCLE guidelines [34]. Electronic databases of MEDLINE (via PubMed), EMBASE and Web of Science were searched for relevant literature up to and including December 2023; the search strategy for MEDLINE is presented in Supplementary Table 1. Bibliographies of the selected studies and relevant review articles were checked for cross-references, and additional relevant studies were obtained using the Google and Google Scholar search engines. Titles and abstracts of the search-identified studies were screened by two authors (S.S. and C.K.) and full texts of all eligible studies were obtained. Uncertainty in the determination of eligibility was resolved by discussion with the other authors. Two authors (S.S. and C.K.) reviewed the selected full texts independently and final inclusion was based on the aforementioned criteria. Inter-rater reliability was measured using the Cohen’s kappa statistic. A summary of the study selection process is presented in Supplementary Fig. 1.

### Data extraction

Based on full-text screening of the selected studies, the following data was extracted using a standardized, pre-piloted form: author(s), study design, animal characteristics, model type, number of animals/defects, number of procedures, intervention(s), observation time(s), outcome(s), method(s) of outcome evaluation, main findings, and conclusions. Missing data was requested from the authors. Descriptive summaries of studies included were entered into tables. Quantitative radiographic and histomorphometric data was extracted for possible meta-analysis; data were recorded as (or converted into) means and standard deviations (SD) for analysis. If data were only expressed graphically, numerical values were requested from the authors, and if no response was received, a digital ruler software was used to measure graphical data (ImageJ; National Institutes of Health, Bethesda, MD, USA).

### Quality assessment and risk of bias

Reporting quality assessment of all studies will be performed based on a modification of the ARRIVE (Animal Research: Reporting In Vivo Experiments) guidelines [35], regarding relevant items [36]. Compliance with the guidelines was evaluated using a predefined grading system applied to each of the 20 items [37] (Supplementary Table 2). Reporting quality was judged as ‘high’, ‘moderate’ or ‘low’. Risk of bias (RoB) assessment is performed using a modification of SYstematic Review Centre for Laboratory animal Experimentation (SYRCLE) RoB tool for animal studies, and judged as ‘high’, ‘low’ or ‘unclear’ [38] (Supplementary Table 3). Any disagreement between the reviewers during study selection, data extraction, and quality assessment was resolved by discussion and consensus.

### Meta-analysis

A meta-analysis was performed to determine the degree of spontaneous regeneration in acute peri-implant defects of minipigs using STATA Statistical Software 12 (StataCorp LP, College Station, TX, USA) and the DerSimonian and Laird random effects model, assuming a degree of heterogeneity between the individual studies [39]. Only studies which included a control group receiving no treatment, i.e., “sham” or “empty defect” group, and reporting quantitative tomographic or histomorphometric outcomes (BA and BIC) were included in the meta-analysis. Separate analyses were performed for the outcomes BA and BIC in intrabony and supra-alveolar defects. Pooled estimates [effect sizes (ES)] were calculated along with 95% confidence intervals (CI). The I^2^ statistic was used as a measure of heterogeneity across studies, with I^2^ > 75% indicating substantial heterogeneity [39]. A univariate meta-regression analysis was performed to test the effects of different variables on pooled ES for each outcome. Publication bias was assessed via funnel plot asymmetry and Egger’s regression test.

## Results

### Search results

The initial search yielded 96 publications (after removing duplicates) studying bone regeneration around implants in pigs. To limit the search to the focused question, only those studies reporting experimental peri-implant bone defects were considered for inclusion [Cohen’s kappa = 0.857 (95% CI 0.811–0.903)]. Based on further eligibility criteria and full-text review, 21 studies reporting on acute defects (*n* = 16) and chronic ligature induced defects (*n* = 5) were included in the review. Among the latter, two publications were possibly different reports from the same experiment [28, 40], while one study was still in progress at the time of this review, but relevant data was obtained directly from the authors [41]. The primary reason for exclusion was assessment of implant osseointegration rather than peri-implant bone regeneration (Supplementary Table 4).

### Study characteristics

All studies reported the use of minipigs, mostly of the Göttingen type. On average, the animals were mostly females, aged 20.38 *±* 4.5 months. The most common anatomical site for defects was the mandibular or maxillary alveolar ridge (premolar-molar region) with a “split-mouth” design (bilateral defects); other sites included the mandibular inferior body. Most studies reported an intraoral surgical approach whereby molar and/or premolar teeth were first extracted, followed by a healing phase, after which standard implants were inserted (mean diameter 3.78 *±* 0.42 mm, mean height 9.55 *±* 1.86 mm). All but two studies [28, 40] reported the use of implants with modified/rough surfaces, most commonly sand-blasted and acid-etched. Based on defect type, the included studies were categorized under ‘acute’ and ‘chronic’ defects (Tables 1 and 2).

**Table 1:** Summary of studies reporting acute peri-implant defects

Year	Study	*N*	Age (m)	Site	*n* /side	Uni / Bi	Implant size (mm)	Implant surface (manufacturer)	Defects - type, size (mm)	Class^#^	Time	Methods	Outcomes
**- Intrabony defects**													
2009	Neugebauer et al. ** (43)	6	18–21	P	5	?	3.8 × 5	SA (Dentsply)	D, 4.2 × 3.5	I-a	4 m	Hm	BA, BIC
2012	Zambon et al. (44)	12	20	P, M1	2	B	4.1 × 8	SA-A (Straumann)	D, 12 × 6 × 2	I-a/b	5 m	Hm	BIC
2014	Friedmann et al. (45)	6	18	P, M1	2	B	4.1 × 8	SA/SA-A (Straumann)	C-D, 5 × 5	I-a/b	4 w	Hm	BA, BIC
2015	Kim et al. (46)	6	24	P	4	B	3 × 10	SA (Osstem)	C, 2 (depth)	I-e	4–12 w	Hm	BA, BIC
2016	Verket et al. (47)	5	20	P	2	B	3.3 × 8	SA-A (Straumann)	D, 6 × 10	I-a	3 m	Hm	BA, BIC
2016	von Wilmowsky et al.* (48)	6^T^	18 *±* 4	-	4	U	4.1 × 12	SA (Straumann)	C-D, 4 × 3 × 3	I-b	90 d	Hm	BA, BIC
2017	Kämmerer et al. (49)	15	22	P	1–2	B	4.3 × 12	SA + CaP (Bonit-ex)	C, 7 × 5	I-e	120 d	Hm	BIC
2018	Verket et al. (50)	6	17–19	P	3	B	3.25 × 11.5	SA (Biomet-3i)	C, 6 × 5	I-e	6 w	mCT, Hm	BA, BIC
2019	Wang et al. (51)	14	12	P, M1	3	B	4.1 × 10	NR	D, 12 × 6 × 2	I-a	3, 6 m	mCT	BA
2020	Tan et al. (52)	5	24	P4	1	?	4.1 × 12	SA (Straumann)	C-D, 10 × 12	I-b	6 m	Hm	BA, BIC
2021	Almansoori et al.* (53)	5	12–18	-	3	B	4 × 8.5	SA (Osstem)	C-D, 8 × 2 × 4	I-b	12 w	mCT, Hm	BA, BIC
2021	Thieu et al. (54)	6	27–32	P	2	B	3 × 11	SA + F (Dentsply)	C-D, 8 × 5 × 6	I-c	12 w	mCT, Hm	BA, BIC
**- Supra-alveolar defects**													
2009	Fenner et al. ** (55)	8	NR	P, M	6	U	NR	SA (Dentsply)	CE, 2–8	II	12 m	Hm	BA, BIC
2012	Freilich et al. ^S^ (56)	8	24	P, M1	2	B	4.1 × 9	SA/SA-A (Straumann)	CE, 2.5	II	9 w	Hm	BIC
2013	Catros et al. ^S^ (57)	6	NR	P, M1	2	B	4.1 × 9	SA/SA-A (Straumann)	CE, 2.5	II	8 w	mCT, Hm	BIC
2017	Schorn et al. (58)	12	NR	P, M1	3	B	3.5 × 11	TO (Nobel Biocare)	CE, 5	II	2–12 w	Hm	BA, BIC

N, number of animals, n, number of implants per side, Uni/Bi, unilateral or bilateral (split-mouth), m, months, w, weeks, NR, not reported; BA, bone area, BIC, bone-to-implant contact

All studies performed in the mandibular ridge except (*) mandibular inferior border and (**) maxilla

SA, sand-blasted acid-etched; SA-A, sand-blasted and chemically treated; TO, titanium oxide blasted; CaP, calcium phosphate; F, fluoride; P, proprietary surface treatment

C, circumferential, D, dehiscence, CE, coronal exposure of implant, P, premolars, M, molars

^#^ Classification according to Schwarz et al. [53]

^T^ 3 diabetic and 3 healthy animals

^S^ Modified abutment design to provide “space maintenance”

**Table 2:** Summary of studies reporting ligature-induced peri-implant defects

Year	Study	Type	*N*	Age (m)	Site	*n* /side	Uni / Bi	Implant size (mm)	Implant surface	Healing	Ligatures	Active induction	Post induction	Methods	Defect description
1991	Hickey et al. * (28)	E	2	NR	P	3	B	NR	M (Nobel-pharma)	8 w	4-0 silk	6 w	NR	Clin, XR, Micro	NR
1993	Singh et al. * (40)	T	2	NR	P	3	B	NR	M (Nobel-pharma)	NR	4-0 silk	6 w	NR	Clin, XR, Histo	*≥* 2 threads exposed
2016	Stubinger et al. (59)	E	6	16–17	P	4	B	4 × 8	SA + P (Thommen Medical)	0	4-0 silk	6 w	0	Clin, mCT	1 (lingual) to 3 (buccal) threads exposed
2018	Rodriguez et al. (60)	E, T	6	NR	P	3	B	3.4 × 9	LM/RBT (Biohorizons)	12 w	Metal	12 w	4 w (only in T group)	Clin, Histo	NR
2019	Ramos et al. ** (41)	E, T	8	NR	P, M1	4	B	4 × 10	SLA + CaP (Intra-lock)	8 w	3-0 silk (surgical)	14 w	4	Clin, XR, Micro, mCT	Wide range (complete to no bone loss); mean defect size after 18 w: 3.15 *±* 2.42 mm

N, number of animals, n, number of implants per side, Uni/Bi, unilateral or bilateral (split-mouth), m, months; w, weeks; E, etiopathogenesis; T, treatment, NR, not reported; Clin, clinical; XR, radiography; Histo, histology; mCt, micro-CT; Micro, microbiological

* Possibly different publications from the same experiment

** Study in progress, data obtained directly from authors

All studies performed in the mandibular ridge

M, machined surface; SA, sand-blasted acid-etched; P, proprietary surface treatment; LM, laser microtextured; RBT, resorbable blast textured; CaP, calcium phosphate coating

#### Acute defects

Most studies reported the use of acute type defects, where defects were created at the time of implant placement and the regenerative intervention was immediately applied (Table 1). These defects could be further classified, according to the classification by Schwarz et al. [42], as:

- intrabony defects (*n* = 12 studies, observation time 4–24 weeks) [43–54], where implants were placed at the level of the alveolar crest and defects were surgically created to simulate Class I defects with “well-defined intrabony components” (circumferential and/or dehiscence).
- supra-alveolar defects (*n* = 4 studies, observation time 2–48 weeks) [55–58], where implants were placed supra-crestally, leaving the coronal 2–5 mm of the implant exposed to simulate Class II defects with “horizontal bone loss” [42].

#### Chronic (ligature induced) defects

Five studies assessed the development of chronic peri-implant disease by means of submarginal placement of ligatures in minipigs [28, 40, 59, 60]. One study was in progress at the time of review, but relevant data was directly obtained from the authors [41] (Table 2). All four studies evaluated the etiopathogenesis of experimentally induced peri-implantitis, and three studies additionally evaluated the efficacy of different therapies. Considerable heterogeneity was observed between studies with regards to the induction protocol. Most studies used silk ligatures around osseointegrated implants (8–12 weeks healing) to accumulate plaque and induce disease; in one study, ligatures were placed simultaneously with the implants, i.e., prior to implant osseointegration [59]. Differences were also found with regards to the length of the “active induction phase”, i.e., duration of ligature placement, ranging from 6 weeks [28, 40] up to 12 [60] or even 14 weeks [41]. The duration of the “chronification phase”, i.e., disease progression after ligature removal, which ranged from 0, i.e., no waiting period between ligature removal and assessment [28, 40, 59] to 4 weeks [41, 60].

### Defect/disease development and spontaneous healing

In eight studies reporting acute defects, a control group of “empty” defects receiving no treatment was included and therefore, spontaneous bone regeneration could be assessed. Outcome assessment was performed via micro-CT and/or histology with histomorphometry. Among the studies using ligature models, most studies reported radiographic bone loss, usually based on a non-quantitative description of “implant thread exposure” (Supplementary Table 5). According to studies, bone loss obtained after the induction period is around 2–3 threads, however some of the studies report a rather random distribution of that bone loss (where some implants developed complete loss of support and others did not experience bone loss) [41]. Three studies used histological analysis to report differences between the treatment groups but without characterizing the defect/disease development, while in two studies, defect development was described based on clinical parameters, mainly probing depth [28, 59]. Two studies provided microbiological data reporting either a shift in the microbiological profile [28] or a significant increase in non-periodontitis related bacteria which was not correlated to the clinical findings [41].

### Meta-analysis

**Fig. 1:**
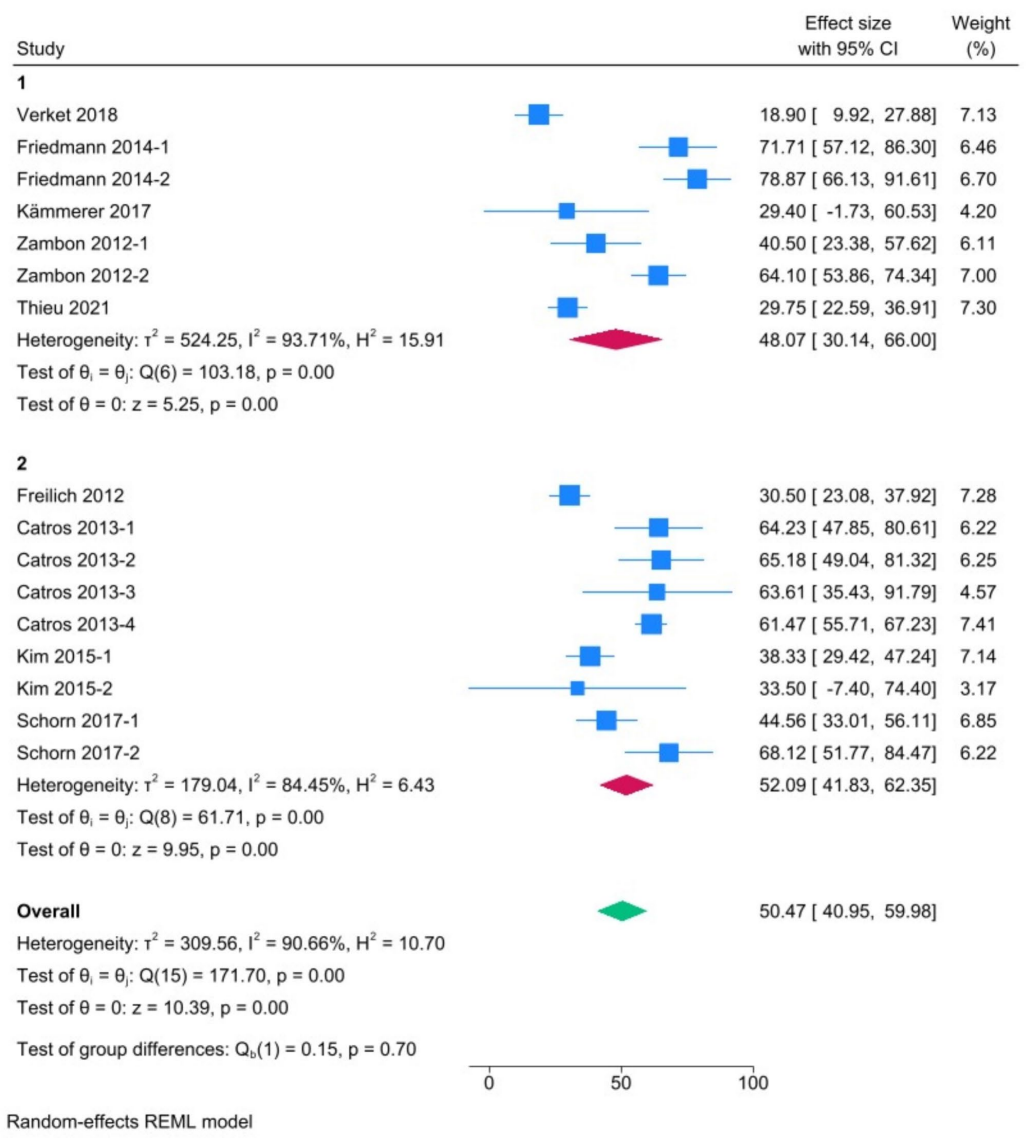
Meta-analysis of studies reporting histomorphometric bone-to-implant-contact (1 = intrabony, 2 = supra-alveolar defects). Results are presented as effect sizes with 95% confidence intervals (CI)

**Fig. 2:**
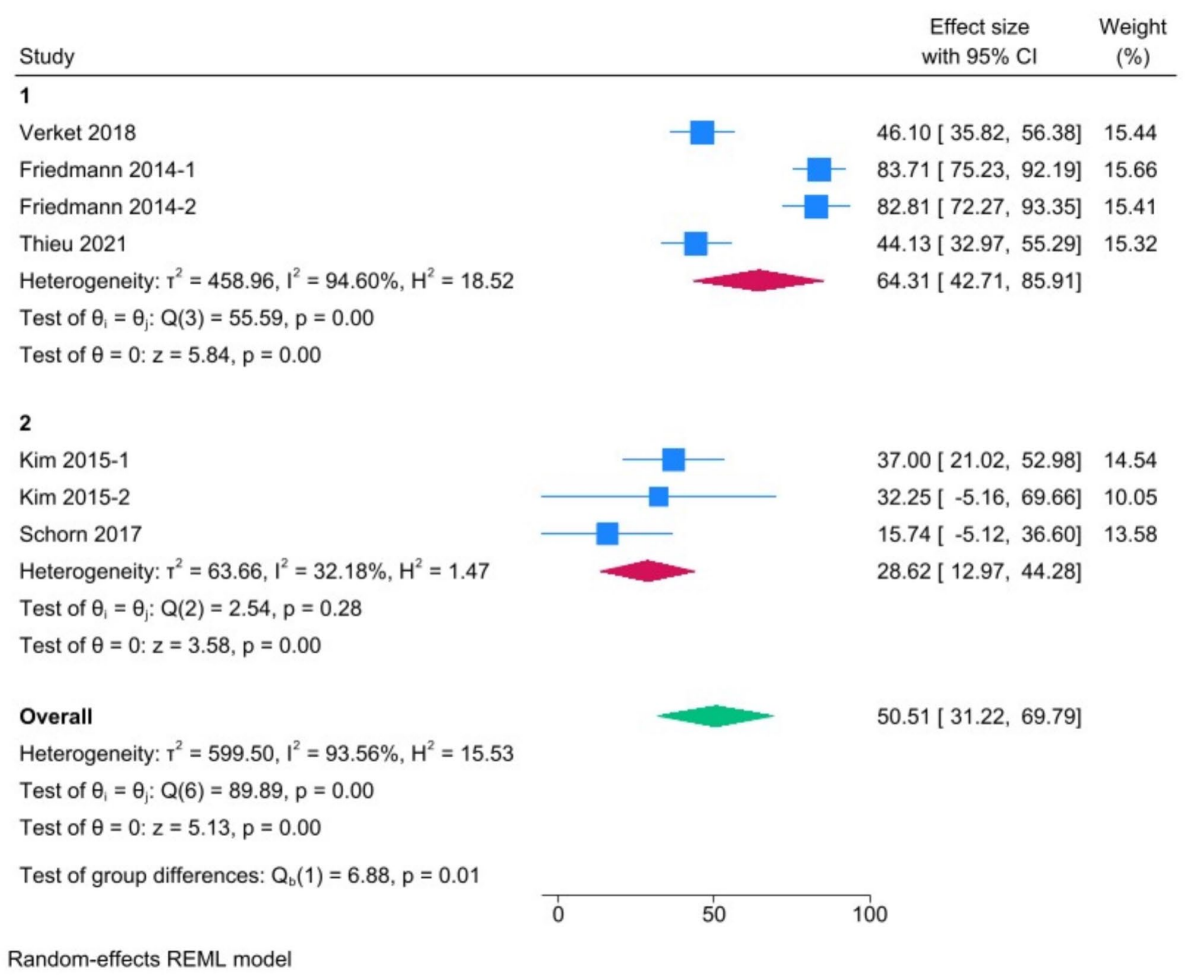
Meta-analysis of studies reporting histomorphometric bone area (1 = intrabony, 2 = supra-alveolar defects). Results are presented as effect sizes with 95% confidence intervals (CI)

A meta-analysis was separately performed for the histomorphometric outcomes BIC (*n* = 9 studies) and BA (*n* = 5 studies); in each case, sub-groups were defined based on defect type, i.e., intrabony and supra-alveolar (Figs. 1 and 2). No meta-analysis could be performed for ligature studies. Overall, the pooled estimates of spontaneous regeneration [ES (95% CI)] were as follows: 48.07% BIC (30.14–66%) and 64.31% BA (42.71–85.91%) in intrabony defects, and 52.09% BIC (41.83–62.35%) and 28.62% BA (12.97–44.28%) in supra-alveolar defects. A univariate meta-regression analysis was performed within each outcome group to test the effect of healing time, but no significant effect was observed (data not shown). All meta-analyses revealed high heterogeneity (I^2^ > 90%), while funnel plot asymmetry and Egger’s tests revealed potentially high publication bias (Supplementary Fig. 2), indicating that the results must be interpreted with caution.

### Quality assessment and risk of bias

The overall quality of the included studies was judged to be average and the RoB was judged to be moderate (Supplementary Tables 6–7). For RoB, the items which most often scored poorly were related to baseline data, housing, blinding of operators, and blinding of assessors. It must be noted that the included studies covered a wide span of publication dates, with many studies being published before the ARRIVE and SYRCLE guidelines. Nevertheless, a clear need for better quality reporting and compliance with these guidelines was identified herein.

## Discussion

The aim of this study was to systematically review the available evidence to identify the most pertinent experimental design using the pig as the experimental animal, for studying the regeneration of peri-implant bone defects. Overall, a modest number of relevant studies (*n* = 21) were identified, mostly with acutely developed defects, demonstrating large heterogeneity in terms of the characteristics of the experimental model used. Most of the experimental defects were created in the mandibular alveolar ridge following extraction of premolars and first molars and after placing standard dental implants. These acute defects could be broadly classified as intrabony (circumferential and/or dehiscence) or supra-alveolar, somewhat simulating human Class I and II peri-implantitis defects [42], respectively. Based on our meta-analysis, this experimental model demonstrated a high capacity for spontaneous bone regeneration (BA) and re-osseointegration (BIC) in both intrabony and supra-alveolar defects. The evidence on the use of chronic ligature induced peri-implantitis (LIPI) in minipigs is limited and does not clearly indicate a natural progression of peri-implantitis in this animal model.

The optimal animal model for evaluating bone regenerative therapies should simulate the clinical scenario by providing a disease profile that is comparable to humans, and allow the use of similar therapies as would be used clinically [61]. Indeed, pigs fulfil these criteria and represent an adequate model of bone regeneration, since they are closely related to humans in terms of bone anatomy, composition, and metabolism, and allow for the use of dental implants and biomaterial scaffolds of clinically relevant dimensions [62]. The studies included in this report have used minipigs, particularly Göttingen minipigs, on average 20 months old, resulting in bone defects morphologically similar to humans. However, in the case of periodontitis and peri-implantitis, an additional relevant criterion for selecting the optimal animal model is the “natural” occurrence of the disease or the possibility to induce the disease, e.g. via placement of ligatures and plaque accumulation, which will “chronically progress” (in terms of continuing bone loss) after removal of the infectious stimulus (ligatures). The LIPI model in dogs [19] is considered as the ‘gold standard’ for investigating both the pathogenesis and therapy of peri-implantitis, given their natural tendency to develop periodontitis and the possibility to induce peri-implantitis with a tendency for natural progression [18, 63]. In contrast, only a few studies have reported the experimental induction of periodontitis using ligatures in pigs [64, 65]. Similarly, only four studies reporting LIPI in pig models were identified in the present review. Considerable heterogeneity was observed among these studies in terms of the experimental protocols used, specifically on the use of different ligature placement and disease induction protocols. Despite differences in the length of the active ligature induced disease period (ranging from 6 to 14 weeks), the degree of bone loss achieved did not vary remarkably between the studies. Moreover, unlike in dogs, the configurations of resulting bone defects did not frequently resemble naturally occurring lesions in humans. Despite differences in the radiographic assessment methods among the studies, the attained bone loss was often restricted to the first 2 or 3 threads and its occurrence was unpredictable. Indeed, in some studies there was no consistent bone loss pattern (some of the implants had exhibited complete supporting bone loss, while others in the same study or animal showed absence of bone loss after 14 weeks of active induction period) [60]. Therefore, based on limited evidence, it appears that bone defects from ligature placement in pigs occur as a result of mechanical trauma rather than infectious disease progression.

It is of relevance to discuss the nature of peri-implant bone defects in pigs in the context of corresponding defects observed in other animal models, most commonly dogs, and in humans. The induction of experimental chronic peri-implantitis lesions, via the placement of ligatures (LIPI), is well established in the dog model. Several studies have demonstrated that the initiation and progression of LIPI in dogs follows a similar pattern as in humans, as evidenced by a “spontaneous progression” phase following the removal of ligatures (“active breakdown” phase) [18, 63]. Moreover, Schwarz et al. [42] reported that the configurations and sizes of LIPI bone defects in dogs resemble naturally occurring peri-implantitis lesions in humans; circumferential defects associated with a horizontal alveolar bone loss, were most frequently observed in both dogs (86.6%) and humans (55.3%). However, in a recent systematic review of canine LIPI models (*n* = 36 studies), Solderer et al. [66] reported large variations in defect ‘depth’ measurements across studies and over time (defect ‘morphologies’ were not considered in this review). More recently, a classification of peri-implantitis defects was presented by Monje et al. [4] based on CBCT data from human implant sites. Contrary to previous reports, the authors found that the most common defect morphology at the patient- (87%) and implant-level (55%) was an “infraosseous 2–3 wall defect”, frequently including a component of buccal bone loss (22%). Other studies have reported predominantly non-circumferential buccal dehiscence (34%) [67] or circumferential bone loss with or without buccal dehiscence (25–30%) [68] in human peri-implantitis lesions. In context, LIPI bone defects in pigs do not seem to demonstrate any predictable patterns or resemble those configurations frequently encountered in human peri-implantitis lesions. Given that bone defects from ligature placement seemed to occur as a result of mechanical trauma rather than infectious disease progression, even the LIPI in pigs can be considered as “acute” defects. Further well-designed studies are needed to characterize and compare the morphologies of LIPI bone defects in pigs.

In light of the difficulties associated with the use of the chronic LIPI model, in addition to the extended time and high costs, most of the included studies used acute bone defects, surgically created around implants to mimic defect configurations naturally occurring peri-implantitis lesions; the regenerative intervention is then applied in the same surgical session. In a majority of the identified studies the resulting bone defects are either Class I or intrabony or defects, most frequently with a combined circumferential-dehiscence configuration (Class I-c/d) according to Schwarz et al. [42]. While acute type defects may be less time consuming and easier to implement than LIPI, major limitations of this approach are as follows. Firstly, the absence of a microbial/infectious component, which is the primary challenge in peri-implantitis progression and treatment, and secondly, the potential bias introduced by spontaneous regeneration of these defects depending on the animal model; a high degree of spontaneous regeneration may confound the detection of clinically meaningful effects of the tested therapy. Indeed, a high degree of spontaneous regeneration was observed in our meta-analysis in terms of histomorphometric BIC and BA (~ 50%), especially in intrabony defects. Even in supra-alveolar defects, i.e., when the coronal portion of the implant was left exposed/unsubmerged, the pooled BIC was 52.09% (95% CI: 41.83–62.35%), suggesting that there was substantial new bone growth along the exposed implant surface outside the original bony envelope [it must be noted that in two studies of supra-alveolar implant placement, a modified “umbrella” abutment design was used to function as a “scaffold retainer”, which provided some degree of space maintenance and possibly primary clot stability to facilitate healing [56, 57]]. Not surprisingly, these values correlate closely with our recent meta-analysis of spontaneous bone regeneration in experimental alveolar defects in pigs, which was also found to be ~ 40–50% [31]. Even critical-sized defects that were allowed to become chronic in minipigs showed a higher degree of spontaneous regeneration compared to similar defects in dogs [31]. This may be attributed to differences in bone metabolism/healing rates between species [26]. The spontaneous regeneration phenomenon is not unique to minipigs. Indeed, even in ‘gold standard’ canine LIPI defects, a ‘self-arresting’ phase often occurs after the active breakdown phase, i.e., removal of ligatures, and may even result in some ‘recovery’ (bone regeneration) before further disease progression [63]. However, in minipigs, this innate healing capacity may be especially high, which further limits the feasibility of LIPI since, as previously mentioned, if the lesions were allowed to become chronic, the defects would likely self-resolve. Thus, given the limitations of acute defects and the challenges associated with establishing LIPI, the appropriateness of the minipig model for peri-implantitis research may be questioned.

Some limitations of the present review must be acknowledged. Firstly, the quality of the included studies was judged to be moderate and a large heterogeneity was observed in terms of the experimental settings, which was reflected in the meta-analyses and which affects the overall quality of the synthesized evidence. Secondly, a wide range of observation (and ‘disease induction’) times for both acute and chronic type defects was observed across the included studies. Indeed, longer observation times may reveal greater BA and BIC values. Although a univariate regression analysis revealed no significant effect of time on BA or BIC in acute defects, possibly due to heterogeneous data, the ‘time’ variable may have introduced some bias in the analysis. Finally, the influence of implant properties (macro-/micro-geometry, surface characteristics, coatings, etc.), known to influence the progression/ outcomes of peri-implantitis and capacity for re-osseointegration [69, 70], were rarely considered among the included studies and might have influenced the results. Nevertheless, based on the reviewed literature, the following factors may be considered when selecting an experimental peri-implantitis animal model;

- In pigs, the use of acute peri-implant defects may be questioned due to the limited clinical relevance of the method (absence of microbial insult) and high capacity for spontaneous regeneration.
- The feasibility of inducing peri-implantitis via ligatures is also questionable due to the lack of convincing data demonstrating establishment of a pathological microbial milieu (correlated with clinical findings), and evidence of continual disease progression after ligature removal, as has been described in dogs.
- Consequently, dogs may still represent the preferred animal model for experimental peri-implantitis pathogenesis and therapy.

## Conclusions

Based on our inclusion criteria, we identified 21 studies evaluating bone regeneration in experimental peri-implant defects in pigs. The results are derived mainly from acute defects in adult female Göttingen minipigs, which could be broadly classified as intrabony (combined circumferential and dehiscence defects) or supra-alveolar (horizontal) defects. Evidence for chronic LIPI in this animal model is inconclusive; bone defects from ligature placement occur most likely as a result of mechanical trauma rather than infectious disease progression. Until further well-designed studies demonstrate the feasibility of inducing LIPI with spontaneous progression in pigs, dogs represent the preferred animal model for experimental peri-implantitis.

## Electronic supplementary material

Below is the link to the electronic supplementary material.

Supplementary Material 1

## Data Availability

No datasets were generated or analysed during the current study.

## Declarations

### Registration

The review was prospectively registered on PROSPERO: International Prospective Register of Systematic Reviews database (CRD42023450700).
