# Supplementary material for: Peri-implant bone regeneration in pigs
Source: Int J Implant Dent. 2024 Nov 15;10:55. doi: 10.1186/s40729-024-00572-9 (PMC11568104; doi:10.1186/s40729-024-00572-9)
Supplement: Supplementary file 1 — Supplementary Material 1 [file 40729_2024_572_MOESM1_ESM.docx]

**Supplementary file**

**Supplementary table 1: Search strategy for MEDLINE**

| Ovid MEDLINE(R) and Epub Ahead of Print, In-Process, In-Data-Review & Other Non-Indexed Citations and Daily <1946 to August 17, 2023> |
| --- |
| 1 Peri-Implantitis/ 2095  2 (periimplantitis or "peri implantitis" or peri-implant or periimplant or "peri implant").ti,ab,kf. 11139  3 1 or 2 11250  4 Bone Regeneration/ 18427  5 (bone adj2 (regeneration* or regenerative or graft* or substitute* or augmentation* or engineering or defect*)).ti,ab,kf. 73149  6 4 or 5 79355  7 sus scrofa/ or swine, miniature/ 23201  8 (pig or pigs or minipig* or "mini pig*" or mini-pig* or "miniature pig*" or micropig* or "micro pig*" or micro-pig* or swine or swines or "miniature swine*" or miniature-swine* or "Sus scrofa" or "Sus domestica" or porcine).ti,ab,kf. 330622  9 7 or 8 334198  10 3 and 6 and 9 85 |
| Total: 85 |

**Supplementary table 2: Checklist for quality assessment of studies according to modified ARRIVE guidelines (Kilkenny et al. 2010) and a predefined grading system (Schwarz et al. 2012)**

| **Item** | **Description** | **Grade** |
| --- | --- | --- |
| 1 | Title | 0 = inaccurate/not concise  1 = accurate and concise |
| 2 | Abstract  Summary of the background, research objectives, including details of the species or strain of animal used, key methods, principal findings and conclusions of the study | 0 = clearly inaccurate  1 = possibly accurate  2 = clearly accurate |
| 3 | Introduction  Background – objectives, experimental approach and rationale, relevance to human biology | 0 = clearly insufficient  1 = possibly sufficient  2 = clearly sufficient |
| 4 | Introduction  Objectives – primary and secondary | 0 = not clear  1 = clear |
| 5 | Methods  Ethical statement – nature of the review permission, relevant licences, national and institutional guidelines for the care and use of animals | 0 = clearly insufficient  1 = possibly sufficient  2 = clearly sufficient |
| 6 | Methods  Study design (a) number of experimental and control groups; (b) inclusion of an untreated control group; and (c) steps taken to minimize bias (blinding of assessors) | 0 = no  1 = yes (x3) |
| 7 | Methods  Experimental procedure – precise details (i.e. how, when, where, why) | 0 = clearly insufficient  1 = possibly sufficient  2 = clearly sufficient |
| 8 | Methods  Experimental animals – species, strain, sex, developmental stage, weight, source of animals | 0 = clearly insufficient  1 = possibly sufficient  2 = clearly sufficient |
| 9 | Methods  Housing and husbandry – conditions and welfare-related assessments and interventions (i.e. type of cage, bedding material, number of cage companions, light/dark cycle, temperature, access to food and water) | 0 = clearly insufficient  1 = possibly sufficient  2 = clearly sufficient |
| 10 | Methods  Sample size – total number of animals used in each experimental group, details of calculation | 0 = clearly inadequate  1 = possibly adequate  2 = clearly adequate |
| 11 | Methods  Allocation animals to experimental groups – randomization or matching, order in which animals were treated and assessed | 0 = no  1 = yes |
| 12 | Methods  Experimental outcomes – definition of primary and secondary outcomes | 0 = no  1 = unclear/not complete  2 = yes |
| 13 | Methods  Statistical methods – details and unit of analysis | 0 = no  1 = unclear/not complete  2 = yes |
| 14 | Results  Baseline data – characteristics and health status of animals | 0 = no  1 = yes |
| 15 | Results  Numbers analysed – absolute numbers in each group included in each analysis, explanation for exclusion | 0 = clearly inadequate  1 = possibly adequate  2 = clearly adequate |
| 16 | Results  Outcomes and estimation – results for each analysis with a measure of precision, as standard error or confidence interval | 0 = no  1 = unclear/not complete  2 = yes |
| 17 | Results  Adverse events – details and modifications for reduction | 0 = no  1 = unclear/not complete  2 = yes |
| 18 | Discussion  Interpretation/scientific implications – study limitations including animal model, implications for the 3Rs (replacement, refinement or reduction) | 0 = clearly inadequate  1 = possibly adequate  2 = clearly adequate |
| 19 | Discussion  Generalisability/translation – relevance to human biology | 0 = clearly inadequate  1 = possibly adequate  2 = clearly adequate |
| 20 | Discussion  Funding – sources, role of the funders | 0 = clearly inadequate  1 = possibly adequate  2 = clearly adequate |

**Supplementary table 3: Assessment of risk of bias in included studies using the modified SYRCLE tool (Yan et al. 2015)**

| **Risk of bias domain (response for each: yes/no/unclear)** |
| --- |
| 1. Was the allocation sequence adequately generated and applied?  2. Were the groups similar at baseline or adjusted for confounders?  3. Was the allocation adequately concealed?  4. Were the animals randomly housed during the experiment?  5. Were the caregivers and/or investigators blinded?  6. Were animals selected at random for outcome assessment?  7. Was the outcome assessor blinded?  8. Were incomplete outcome data adequately addressed?  9. Was it stated that the experiment was randomized at any level?  10. Was it stated that the experiment was blinded at any level? |

**Supplementary table 4: List of studies excluded after full-text review**

| **Year** | **Study** | **Reason for exclusion** |
| --- | --- | --- |
| 2011 | Hasturk et al. [1] | Implants placed in extraction sockets |
| 2020 | Karl et al. [2] | No peri-implant defects |
| 2018 | Kim et al. [3] | In vitro study |
| 2011 | Kotenko et al. [4] | Implants placed in extraction sockets |
| 2020 | Lyu et al. [5] | No peri-implant defects |
| 2016 | Mir Mari et al. [6] | In vitro study |
| 2017 | Mir Mari et al. [7] | In vitro study |
| 2011 | Ogunsalu et al. [8] | No peri-implant defects |
| 2023 | Parvini et al. [9] | Implants placed in extraction sockets |

**Supplementary Table 5: Summary of outcome measures reported in ligature studies**

| Study | Outcome variables | Bone loss | Clinical | Microbiological variables | Histological variables | Immunological variables |
| --- | --- | --- | --- | --- | --- | --- |
| Hickey 1991 | XR (periapical),  Clinical (PD, CAL, inflammation), Microbiological (Culture) | N/a | Slight increase in clinical variable in the induction group compared to control: AL: 5.17 – 6.25 / Control: stable: 5.21 - 5.2  PD: Test: 3.45-4.45 / control 3.87-4.12  GI: Test: 1.58 - 2 / Control: 1.79 - 1.2  PI: Test: 2.12-3 / 2.54 - 2.54 | Test: Gram- species predominantly and increased proportions of spirochetes 12%-35% / control Gram+ species predominantly | N/a | N/a |
| Singh 1993 | Clinical (intra-surgical measurement of defects)  Histological | "osseous defects confirmed clinically and radiographically" At least 2 threads exposed | N/a | N/a | Only in SEM and related to comparative between therapy groups (re-osseointegration) | N/a |
| Stubinger 2016 | Clinical (PD, CAL, inflammation), Micro-CT | Micro-CT: Test: "Buccal bone height reduced up to three implant threads, lingual only one implant thread. Control: No bone loss detected | PD: Healthy 2.2 + 1.1 mean mm / test: 5.4 + 1.9  GI: Control: slight bleeding in 40% of the cases, test: 90% severe bleeding | N/a | N/a | N/a |
| Rodriguez 2018 | Clinical (observations), Histological | N/a | N/a | N/a | Soft tissue: animals post breakdown had higher soft tissue height 3.8+0.4 than post treatment animals 1.838+1.5. Bone loss only related to comparative between post therapy groups | N/a |
| Ramos (in progress) | Clinical, XR, Microbiological (DNA-DNA Checkerboard), Immunological (qualitative and quantitative IL6,8,1B), Micro-CT, Histomorphometry, clinical measurement of defects (flap reopening) | Random bone loss (in some implants complete bone loss, in some implants absence of bone loss). Mean defect size after 18 weeks: 3.15 + 2.42 / i-prox: 3.42 + 2.63 | N/a | Significant increase in the following bacteria: A. odontolyticus, C. showae, E. nodatum, F. nucleatum, N. mucosa y C. gingivalis. Some changes were noted, but not directly related with periodontal disease or peri-implant disease per se. | Only comparative measurements between treatment groups, no differences between them | No significant differences between pre-induction and induction periods. Immunological data not consistently related with disease. Note related with human data. e.g. reduction in IL-1B levels throughout induction process |

XR, radiographs; PD, probing depth; CAL, clinical attachment level; GI, gingival inflammation; i-prox, interproximal; N/a, not available

**Supplementary table 6: Quality assessment of the included studies (ARRIVE)**

|  | **Introduction** | | | | **Methods** | | | | | | | | | | | **Results** | | | | **Discussion** | | |
| --- | --- | --- | --- | --- | --- | --- | --- | --- | --- | --- | --- | --- | --- | --- | --- | --- | --- | --- | --- | --- | --- | --- |
| **Study** | **1** | **2** | **3** | **4** | **5** | **6a** | **6b** | **6c** | **7** | **8** | **9** | **10** | **11** | **12** | **13** | **14** | **15** | **16** | **17** | **18** | **19** | **20** |
| Almansoori et al. | 1 | 2 | 1 | 1 | 1 | 1 | 0 | 0 | 0 | 2 | 0 | 1 | 0 | 1 | 1 | 0 | 1 | 1 | 2 | 0 | 0 | 2 |
| Catros et al. | 1 | 2 | 2 | 1 | 1 | 1 | 1 | 0 | 1 | 0 | 0 | 1 | 1 | 2 | 1 | 0 | 1 | 2 | 2 | 1 | 1 | 2 |
| Fenner et al. | 1 | 2 | 1 | 1 | 1 | 1 | 0 | 0 | 1 | 0 | 0 | 1 | 0 | 2 | 1 | 0 | 2 | 2 | 1 | 1 | 1 | 1 |
| Freilich et al. | 1 | 2 | 2 | 1 | 1 | 1 | 1 | 0 | 1 | 1 | 0 | 1 | 0 | 1 | 1 | 0 | 1 | 1 | 2 | 1 | 1 | 1 |
| Friedmann et al. | 1 | 1 | 1 | 1 | 0 | 1 | 1 | 1 | 1 | 1 | 0 | 1 | 1 | 1 | 2 | 0 | 1 | 2 | 2 | 1 | 1 | 1 |
| Kämmerer et al. | 1 | 2 | 2 | 1 | 1 | 1 | 1 | 1 | 1 | 1 | 0 | 1 | 1 | 2 | 1 | 0 | 1 | 2 | 0 | 1 | 1 | 2 |
| Kim et al. | 1 | 1 | 2 | 1 | 0 | 1 | 1 | 0 | 1 | 1 | 0 | 0 | 0 | 1 | 1 | 0 | 0 | 1 | 0 | 1 | 1 | 0 |
| Le Thieu et al. | 1 | 2 | 2 | 1 | 1 | 1 | 1 | 0 | 1 | 2 | 1 | 0 | 0 | 2 | 1 | 0 | 0 | 2 | 2 | 1 | 1 | 2 |
| Neugebauer et al. | 1 | 1 | 2 | 1 | 1 | 1 | 1 | 0 | 1 | 1 | 0 | 1 | 0 | 1 | 1 | 0 | 1 | 1 | 1 | 1 | 1 | 0 |
| Schorn et al. | 0 | 1 | 2 | 1 | 1 | 1 | 1 | 0 | 1 | 1 | 0 | 0 | 0 | 1 | 1 | 0 | 0 | 1 | 0 | 1 | 1 | 1 |
| Tan et al. | 1 | 2 | 2 | 1 | 0 | 1 | 0 | 0 | 1 | 1 | 0 | 1 | 0 | 2 | 0 | 0 | 1 | 1 | 2 | 1 | 1 | 0 |
| Verket et al. | 1 | 2 | 2 | 1 | 1 | 1 | 0 | 0 | 1 | 2 | 0 | 1 | 0 | 2 | 0 | 0 | 1 | 2 | 2 | 1 | 1 | 2 |
| Verket et al. | 1 | 2 | 2 | 1 | 1 | 1 | 1 | 0 | 1 | 2 | 0 | 1 | 1 | 2 | 1 | 0 | 1 | 1 | 2 | 1 | 1 | 1 |
| Von Wilmonsky et al. | 1 | 2 | 2 | 1 | 2 | 1 | 0 | 1 | 1 | 0 | 1 | 1 | 0 | 2 | 1 | 0 | 1 | 1 | 1 | 1 | 1 | 2 |
| Wang et al. | 1 | 1 | 1 | 1 | 0 | 1 | 1 | 0 | 1 | 1 | 0 | 0 | 1 | 1 | 0 | 0 | 0 | 1 | 1 | 0 | 0 | 2 |
| Zambon et al. | 1 | 2 | 2 | 1 | 1 | 1 | 1 | 0 | 1 | 1 | 0 | 1 | 1 | 2 | 2 | 0 | 1 | 2 | 2 | 1 | 1 | 1 |
| Rodriguez et al. | 1 | 2 | 1 | 1 | 2 | 1 | 0 | 1 | 2 | 1 | 0 | 1 | 1 | 1 | 1 | 1 | 1 | 1 | 1 | 1 | 0 | 2 |
| Singh et al. | 1 | 1 | 1 | 1 | 2 | 1 | 1 | 0 | 1 | 1 | 0 | 0 | 0 | 1 | 0 | 1 | 0 | 0 | 1 | 0 | 0 | 0 |
| Stubinger et al. | 0 | 1 | 1 | 0 | 1 | 1 | 1 | 0 | 1 | 2 | 2 | 1 | 0 | 0 | 0 | 0 | 0 | 0 | 0 | 0 | 1 | 2 |
| Hickey et al. | 1 | 1 | 1 | 1 | 0 | 1 | 1 | 0 | 1 | 1 | 0 | 0 | 0 | 1 | 0 | 1 | 0 | 0 | 0 | 0 | 0 | 1 |
| Ramos et al. | 1 | 1 | 1 | 1 | 0 | 1 | 0 | 0 | 2 | 1 | 0 | 1 | 1 | 1 | 0 | 1 | 1 | 1 | 1 | 0 | 0 | 0 |

0 = clearly inaccurate; 1 = possibly accurate; 2 = clearly accurate

**Supplementary table 7: Risk of bias assessment in the included studies (SYRCLE)**

|  | **Risk of Bias domain** | | | | | | | | | |
| --- | --- | --- | --- | --- | --- | --- | --- | --- | --- | --- |
| **Study** | **1** | **2** | **3** | **4** | **5** | **6** | **7** | **8** | **9** | **10** |
| Almansoori et al. | N | Y | N | U | U | U | U | U | N | N |
| Catros et al. | U | U | U | U | U | U | U | U | Y | N |
| Fenner et al. | N | U | N | U | U | U | U | Y | N | N |
| Freilich et al. | N | U | N | U | U | U | U | U | N | N |
| Friedmann et al. | U | U | U | U | Y | U | U | U | Y | Y |
| Kämmerer et al. | Y | U | U | U | U | U | Y | U | Y | Y |
| Kim et al. | N | U | N | U | U | U | U | U | N | N |
| Le Thieu et al. | N | Y | N | U | U | U | U | U | N | N |
| Neugebauer et al. | N | U | N | U | U | U | U | U | N | N |
| Schorn et al. | N | U | N | U | U | U | U | U | N | N |
| Tan et al. | N | U | N | U | U | U | U | U | N | N |
| Verket et al. | N | Y | N | U | U | U | U | U | N | N |
| Verket et al. | U | Y | U | U | U | U | U | U | Y | N |
| Von Wilmonsky et al. | N | U | N | U | U | U | Y | U | N | Y |
| Wang et al. | U | U | U | U | U | U | U | U | Y | N |
| Zambon et al. | Y | U | U | U | U | U | U | U | Y | N |
| Rodriguez et al. | Y | Y | U | U | N | Y | Y | U | Y | Y |
| Singh et al. | N | U | N | U | N | U | U | U | N | N |
| Stubinger et al. | U | U | N | U | N | U | N | U | U | N |
| Hickey et al. | N | U | N | U | N | U | U | U | N | N |
| Ramos et al. | Y | Y | U | U | U | U | U | U | Y | U |

Y, yes; N, no; U, unclear

**Supplementary Figure 1: PRISMA flowchart for study selection**

**Identification of studies via other methods**

**Identification of studies via databases and registers**

Records identified from citation searching (n = 11)

Records removed *before screening*:

Duplicate records (n = 4)

Records identified from electronic databases (n = 85)

**Identification**

Records excluded:

In vitro studies, non-alveolar sites, osseointegration studies

(n = 62)

Records screened:

Title/abstract (n = 81)

Reports not retrieved

(n = 0)

Reports sought for retrieval

(n = 11)

Reports sought for retrieval:

Full text (n = 19)

Reports not retrieved

(n = 0)

**Screening**

Reports excluded:

Immediate implants in extraction sockets, in vitro studies, osseointegration studies (n = 9)

Reports excluded

(n = 0)

Reports assessed for eligibility

(n = 11)

Reports assessed for eligibility

(n = 19)

Studies included in review

(n = 21)

**Included**

*From:* Page MJ, McKenzie JE, Bossuyt PM, Boutron I, Hoffmann TC, Mulrow CD, et al. The PRISMA 2020 statement: an updated guideline for reporting systematic reviews. BMJ 2021;372:n71. doi: 10.1136/bmj.n71. For more information, visit: <http://www.prisma-statement.org/>

**Supplementary Figure 2: Funnel plots for the outcomes bone-to-implant contact (A) and bone area (B)**

**A.**

**
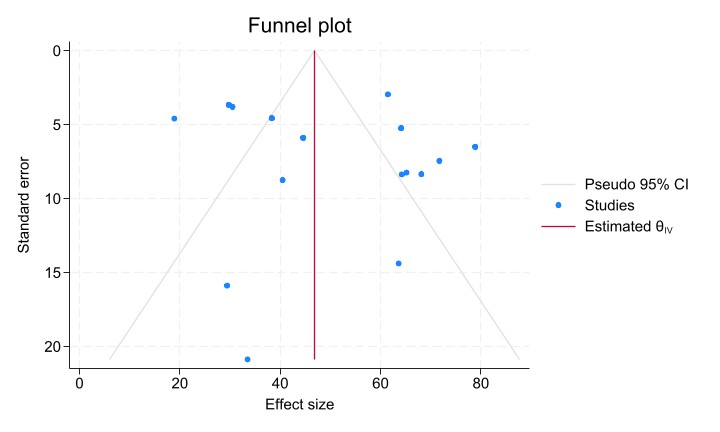
**

**B.**

**
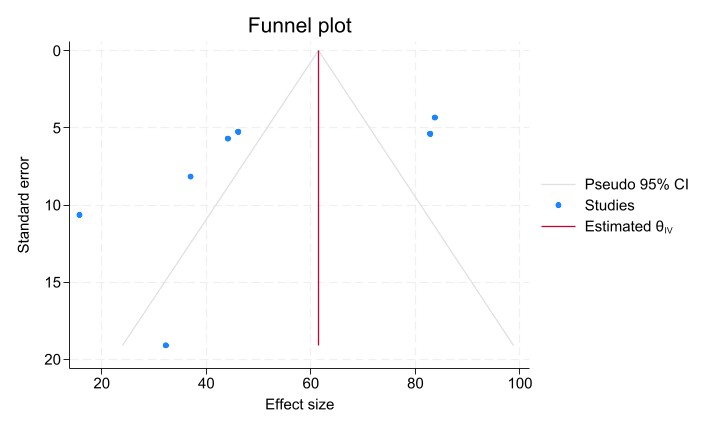
**

**Supplementary references**

1. Hasturk, H., et al., *The use of light/chemically hardened polymethylmethacrylate, polyhydroxyethylmethacrylate, and calcium hydroxide graft material in combination with polyanhydride around implants in minipigs: part I: immediate stability and function.* J Periodontol, 2011. **82**(9): p. 1339-52.

2. Karl, M., et al., *A Pilot Animal Study Aimed at Assessing the Mechanical Quality of Regenerated Alveolar Bone.* Int J Oral Maxillofac Implants, 2020. **35**(2): p. 313-319.

3. Kim, S., et al., *Quantitative measurement of peri-implant bone defects using optical coherence tomography.* Journal of periodontal & implant science, 2018. **48**(2): p. 84-91.

4. Kotenko, M.V. and L.L. Meysner, *Morphological features of peri-implant tissue after placement of dental implants into the extraction socket.* Bulletin of experimental biology and medicine, 2011. **151**(4): p. 492-7.

5. Lyu, H.Z. and J.H. Lee, *The efficacy of rhBMP-2 loaded hydrogel composite on bone formation around dental implants in mandible bone defects of minipigs.* Biomater Res, 2020. **24**: p. 5.

6. Mir-Mari, J., et al., *Influence of blinded wound closure on the volume stability of different GBR materials: an in vitro cone-beam computed tomographic examination.* Clin Oral Implants Res, 2016. **27**(2): p. 258-65.

7. Mir-Mari, J., et al., *Influence of wound closure on the volume stability of particulate and non-particulate GBR materials: an in vitro cone-beam computed tomographic examination. Part II.* Clin Oral Implants Res, 2017. **28**(6): p. 631-639.

8. Ogunsalu, C., et al., *Comparative study of osteoblastic activity of same implants (Endopore) in the immediate extraction site utilizing single photon emission computerized tomography: peri-implant autogeneous bone grafting with GTR versus no peri-implant bone grafting--experimental study in pig model.* West Indian Med J, 2011. **60**(3): p. 336-9.

9. Parvini, P., et al., *Influence of loading and grafting on hard- and soft-tissue healing at immediately placed implants: An experimental study in minipigs.* Journal of clinical periodontology, 2023. **50**(2): p. 232-241.
